# Association between Social Anxiety and Visual Mental Imagery of Neutral Scenes: The Moderating Role of Effortful Control

**DOI:** 10.3389/fpsyg.2017.02323

**Published:** 2018-01-11

**Authors:** Jun Moriya

**Affiliations:** Faculty of Sociology, Kansai University, Osaka, Japan

**Keywords:** social anxiety, mental imagery, vividness, visual imagery, effortful control

## Abstract

According to cognitive theories, verbal processing attenuates emotional processing, whereas visual imagery enhances emotional processing and contributes to the maintenance of social anxiety. Individuals with social anxiety report negative mental images in social situations. However, the general ability of visual mental imagery of neutral scenes in individuals with social anxiety is still unclear. The present study investigated the general ability of non-emotional mental imagery (vividness, preferences for imagery vs. verbal processing, and object or spatial imagery) and the moderating role of effortful control in attenuating social anxiety. The participants (*N* = 231) completed five questionnaires. The results showed that social anxiety was not necessarily associated with all aspects of mental imagery. As suggested by theories, social anxiety was not associated with a preference for verbal processing. However, social anxiety was positively correlated with the visual imagery scale, especially the object imagery scale, which concerns the ability to construct pictorial images of individual objects. Further, it was negatively correlated with the spatial imagery scale, which concerns the ability to process information about spatial relations between objects. Although object imagery and spatial imagery positively and negatively predicted the degree of social anxiety, respectively, these effects were attenuated when socially anxious individuals had high effortful control. Specifically, in individuals with high effortful control, both object and spatial imagery were not associated with social anxiety. Socially anxious individuals might prefer to construct pictorial images of individual objects in natural scenes through object imagery. However, even in individuals who exhibit these features of mental imagery, effortful control could inhibit the increase in social anxiety.

## Introduction

Cognitive theories suggest that socially anxious individuals generate a negative self-image in social situations, and the negative mental imagery contributes to the maintenance of social anxiety (Clark and Wells, 1995; Rapee and Heimberg, 1997; Hofmann, 2007). They imagine seeing themselves from the perspective of an outside observer and overfocus on the evaluation of others or exhibit physical symptoms such as blushing, sweating, or trembling of hands (Clark and Wells, 1995; Hackmann et al., 2000; Hirsch et al., 2006). Negative imagery increases the use of safety behaviors such as avoiding eye contact, and it exacerbates poor performance in conversation in socially anxious individuals (Hirsch et al., 2003, 2004). Based on these theories and findings, several therapies include an imagery technique (Nilsson et al., 2012; McEvoy and Saulsman, 2014; McEvoy et al., 2015; Reimer and Moscovitch, 2015). For example, in an imagery-enhanced cognitive behavioral group therapy, individuals with social anxiety disorder generated negative imagery and updated it by incorporating new information to create new images (McEvoy and Saulsman, 2014; McEvoy et al., 2015). Therefore, clarifying the features of mental imagery in social anxiety is important for both maintenance mechanisms and treatments.

While many previous studies have investigated negative mental imagery in individuals with social anxiety, few studies have examined the general ability pertaining to non-emotional mental imagery. It is important to focus on the general ability of neutral mental imagery owing to the following two reasons. First, greater neutral mental imagery ability might be associated with efficient treatment. McEvoy et al. (2015) showed that the change scores of social anxiety symptoms were positively associated with the vividness of visual imagery in neutral situations. Second, it is possible that non-emotional cognitive function, and not severe emotional processing, has an effect on individuals with social anxiety. In attentional functions, for example, many studies distinguish the effects of emotional processing and cognitive functions on social anxiety (Moriya and Tanno, 2009; Pacheco-Unguetti et al., 2010; Heeren et al., 2015). Heeren et al. (2015) showed that improved general attentional control, rather than emotional processing, is important for decreasing anxiety symptoms. Thus, investigating the effects of non-emotional mental imagery is important.

When assessing mental imagery in social anxiety, Pearson et al. (2013) proposed general imagery use and experience as a key domain. General imagery use and experience focuses on the phenomenological characteristics or conscious experience of mental imagery using self-report questionnaires. It includes assessment of the vividness and visual imagery vs. verbal processing style. Vividness is determined as the storage of rich, clear, and detailed sensory-based representations held in the visual and auditory systems of the working memory (Baddeley and Andrade, 2000). With respect to their processing style, Paivio (1971) and Richardson (1977) classified individuals into two categories: visualizers (also called imagers) and verbalizers. When attempting to perform cognitive tasks, a visualizer relies primarily on mental pictures, whereas a verbalizer relies primarily on verbal-analytical strategies. Both vividness and the processing style were associated with anxiety. Previous studies have reported the generation of highly vivid visual images of “negative” situations in anxiety and social anxiety (Stöber, 2000; Wild et al., 2007; Morina et al., 2011). Inducing “negative” mental images also increased state anxiety in visual processing to a greater extent compared to that in verbal processing when participants listened to negative descriptions (Holmes and Mathews, 2005).

A few studies have examined general “non-emotional” imagery use and experience. In Moore (2002), patients with generalized anxiety disorder were asked to generate an image of “natural” scenes (e.g., color of clothes, characteristic behavior while walking) and rate the vividness of the image. The results showed high imagery vividness of natural scenes in those with anxiety disorders. Although this result is important for demonstrating the role of general vivid imagery in anxiety, there are few studies of non-emotional mental imagery in anxiety and it is unclear whether “social anxiety” is also associated with vivid imagery for neutral scenes; additionally, the sample size was not adequate in Moore (2002). Moreover, it is also important to examine mental imagery in non-clinical samples. Previous studies have shown that social anxiety disorder exists on a continuum with less severe social anxiety on one end and extreme social anxiety on the other (Kollman et al., 2006; Ruscio, 2010). Based on the social anxiety continuum, the main purpose of the present study was to reveal the association between social anxiety and general non-emotional imagery use and experience in non-clinical individuals.

Another important aspect of visual mental imagery is the processing subsystem for object and spatial imagery. Based on neuropsychological data, Kosslyn (1994, 2005) proposed the theory of visual mental imagery. In this theory, mental images are first generated in the visual buffer, which depicts shape and topographically reconstructs the spatial geometry of objects. Then, an attentional window selects a region of the buffer and sends the pattern of activation to two major subsystems: the object and the spatial pathways (Kosslyn, 2005). The object pathway deals with the recognition of the object, whereas the spatial pathway registers the visual location (Kosslyn et al., 2001). Levine et al. (1985) showed that damage to the object pathway disrupts the ability to visualize objects, whereas damage to the spatial pathway disrupts the ability to visualize locations. Because visual imagery consists of these two distinct subsystems, Blazhenkova and colleagues developed a scale to investigate object and spatial imagery ability (Kozhevnikov et al., 2005, 2010; Blajenkova et al., 2006; Blazhenkova and Kozhevnikov, 2009). In the scale, object imagery refers to the ability to process visual information about objects and scenes in terms of color, shape, brightness, and so on. On the other hand, spatial imagery refers to the ability to process information about spatial relations between objects or their parts and perform spatial transformations.

The main purpose of the present study was to clarify the relationships between social anxiety and general non-emotional imagery use and experience (i.e., vividness, visual imagery and verbal processing, and object-spatial imagery) in non-clinical individuals, using self-report questionnaires. According to a previous study (Moore, 2002), it was hypothesized that imagery vividness was positively correlated with social anxiety. With respect to visual imagery vs. verbal processing style, Holmes and Mathews (2010) suggested that visual imagery, not verbal processing, amplifies anxiety because visual imagery enhances emotional processing by overlapping with perceived events. Therefore, it was hypothesized that social anxiety was not correlated with verbal processing but positively correlated with a preference for visual image processing. Lastly, with respect to object and spatial visual imagery, one of the characteristics of object imagery is vividness (Blajenkova et al., 2006), and Moore (2002) showed a positive association between vividness and anxiety. Therefore, social anxiety might be positively correlated with object visual imagery. On the other hand, spatial imagery requires the individual to change his/her own perspective (i.e., perspective transformation). Individuals with high spatial imagery also accurately succeeded at a mental rotation task (Blajenkova et al., 2006), which is highly correlated with perspective taking to imagine scenes from different orientations of the observer (Hegarty and Waller, 2004). Considering that socially anxious individuals imagine seeing themselves from the observer’s perspective (Rapee and Heimberg, 1997), social anxiety might also be positively correlated with spatial visual imagery.

The other purpose was to reveal the moderator variables that attenuate social anxiety in individuals with maladaptive mental imagery. The present study focused on effortful control (EC; Rothbart et al., 2000, 2011). EC includes the ability to regulate attention voluntarily (i.e., attentional control). Because voluntary attention to a particular aspect of an image is an important factor during mental imagery tasks (Kosslyn et al., 2006), attentional control in EC plays an important role in generating vivid mental imagery. Previous studies have shown the moderating role of EC in attenuating anxiety symptoms and have reported a decrease in anxiety symptoms in individuals with high EC (Stevens et al., 2015). Thus, it was hypothesized that high EC would be able to inhibit the increase of social anxiety even if individuals exhibited maladaptive features of mental imagery.

## Materials and Methods

### Participants

The participants were 231 undergraduates, of whom 162 were women and 69 were men. All participants provided informed consent and completed a questionnaire in a class for course credit. The age of the participants ranged from 18 to 23 years, with a mean age of 19.62 (*SD* = 0.83) years. There were no exclusion criteria in the present study.

### Measures

The participants completed all the questionnaires in the following order.

#### Brief Fear of Negative Evaluation Scale (BFNE; Leary, 1983)

The BFNE scale is a 12-item self-report questionnaire with a 5-point scale ranging from 1 (not at all characteristic of me) to 5 (extremely characteristic of me). It assesses the apprehension or distress caused by others’ negative evaluations, which is a core feature of social anxiety (e.g., I am frequently afraid of other people noticing my shortcomings). The Japanese version of the scale has high internal consistency (α = 0.92) and test–retest reliability for a 3-month interval (*r* = 0.74; Sasagawa et al., 2004). In the present study, its internal consistency was adequately high (α = 0.90).

#### Vividness of Visual Imagery Questionnaire (VVIQ; Marks, 1973)

The VVIQ is frequently used to measure the vividness of visual mental images. It consists of 16 items rated on a 5-point scale, in which participants generate mental images of four natural scenes (e.g., a friend or relative, sunrise, a shop, and the countryside) and are asked to rate the clarity and vividness of the images (e.g., “The precise carriage, length of step, etc., in walking” for a friend or relative scene, “The sky clears and surrounds the sun with blueness” for the countryside scene). Hishitani (2005) translated the scale into Japanese, and the Japanese version has adequate internal consistency (α = 0.78 in the present study).

#### Verbalizer–Visualizer Questionnaire (VVQ; Richardson, 1977)

The VVQ was used to measure the participants’ preference for using imagery (Visualizers) or verbal-logical strategies (Verbalizers) when solving problems. Sunaga and Hanyu (1990) revised the original questionnaire by adding items and conducting a factor analysis. The scale has two factors: the visualizer scale (9 items) and the verbalizer scale (11 items). Both are measured on a 5-point Likert-type scale and exhibit adequate internal consistency (α = 0.77 for the visualizer scale and 0.82 for the verbalizer scale). In the present study, the internal consistency for the visualizer scale was not adequately high (α = 0.66), whereas that for the verbalizer scale was high (α = 0.76). The visualizer scale was positively correlated with the VVIQ, whereas the verbalizer scale was associated with verbal intelligence (Sunaga and Hanyu, 1990).

#### Visual Imagery Style Questionnaire (VISQ; Kawahara and Matsuoka, 2009)

The VISQ is a self-report questionnaire for measuring object imagery and spatial imagery. The object imagery scale refers to the ability to process visual information about objects or scenes (e.g., “When reading a book, I usually form a clear and detailed mental picture that has been described”), whereas the spatial imagery scale refers to the ability to process information about spatial relations between objects or their parts (e.g., “I can easily imagine and mentally rotate 3-dimensional geometric figures”). These two scales are related to object and spatial factors in the Object-Spatial Imagery Questionnaire (OSIQ; Blajenkova et al., 2006). Each scale consists of 12 items on a 5-point scale. The scales of the VISQ have adequate internal consistencies (α = 0.87 for the object imagery scale and 0.85 for the spatial imagery scale) and test–retest reliabilities for a 1-month interval (*r* = 0.75 for the object imagery scale and 0.83 for the spatial imagery scale). In the present study, the internal consistencies were adequately high (α = 0.85 for the object imagery scale and 0.88 for the spatial imagery scale).

#### Effortful Control Scale (EC; Rothbart et al., 2000)

The EC scale consists of 35 items on a 7-point Likert-type scale. The EC scale is a part of the Adult Temperament Questionnaire (Rothbart et al., 2000). The EC scale assesses inhibitory control, activation control, and attentional control. The Japanese version of the EC scale has a high internal consistency (α = 0.90) and test–retest reliability for a 2-week interval (*r* = 0.88; Yamagata et al., 2005). In the present study, internal consistency was adequately high (α = 0.86).

## Results

Descriptive statistics and correlations between the BFNE, VVIQ, VVQ, VISQ, and EC scores have been presented in **Table 1**. The BFNE was positively correlated with the visualizer scale of the VVQ and the object imagery scale of the VISQ, and was negatively correlated with the spatial imagery scale of the VISQ. The VVIQ and verbalizer scale of the VVQ were not significantly correlated with the BFNE. The EC scale was negatively correlated with the BFNE and was positively correlated with the verbalizer scale of the VVQ. The other mental imagery scales were not significantly correlated with the EC scale.

**Table 1:** Summary of means, standard deviations, and correlations among measured variables.

	1	2	3	4	5	6	7
(1) BFNE	–						
(2) VVIQ	0.02	–					
(3) VVQ visualizer	0.15*	0.26**	–				
(4) VVQ verbalizer	0.04	0.06	0.35**	–			
(5) VISQ object	0.15*	0.46**	0.61**	10.24**	–		
(6) VISQ spatial	-0.18**	0.30**	0.16**	0.16*	0.36**	–	
(7) EC	-0.13*	-0.05	0.07	0.23**	0.04**	0.12	–
Mean	42.49	53.19	29.06	31.98	38.62**	27.00	85.65
*SD*	8.33	8.05	4.81	5.90	8.04**	18.78	11.66

*BFNE, Brief Fear of Negative Evaluation scale; VVIQ, Vividness of Visual Imagery Questionnaire; VVQ visualizer, visualizer scale of Verbalizer–Visualizer Questionnaire; VVQ verbalizer, verbalizer scale of Verbalizer–Visualizer Questionnaire; VISQ object, object imagery scale of Visual Imagery Style Questionnaire; VISQ spatial, spatial imagery scale of Visual Imagery Style Questionnaire; EC, Effortful Control scale; ^∗^*p* < 0.05, ^∗∗^*p* < 0.01*.

**FIGURE 1:**
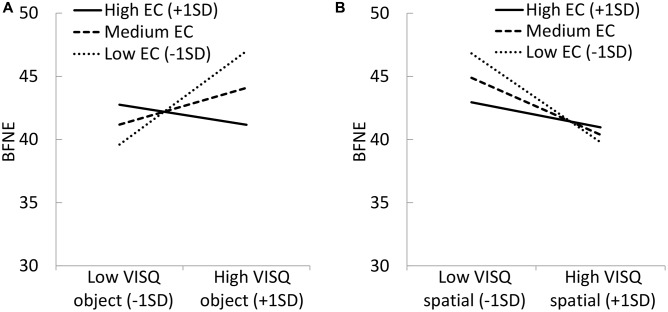
Interactive effects between **(A)** the VISQ object scale and EC, and **(B)** the VISQ spatial scale and EC, on BFNE.

To investigate the interactive effects of the EC scale with the mental imagery scales, hierarchical regression analysis was conducted to predict BFNE scores using the scores on the mental imagery scales and the EC scale. The dependent variable was the BFNE; the independent variables were the VVIQ, visualizer scale of the VVQ, verbalizer scale of the VVQ, object imagery scale of the VISQ, spatial imagery scale of the VISQ; and the moderator variable was the EC scale. First, all independent variables were centered on the grand mean to minimize problems associated with multicollinearity and to scale the variables meaningfully (Aiken and West, 1991; Bauer and Curran, 2005). In Step 1 (main effects), all mental imagery scales and the EC scale were entered, following which, in Step 2 (interaction effect), each mental imagery scale × EC scale interaction was entered. The results of the regression analysis have been shown in **Table 2**. The final two-stage model was significant, *F*(11,219) = 4.12, *p* < 0.001, *R*^2^ = 0.17, *Adj. R*^2^ = 0.13. The object imagery scale of the VISQ was still significantly positively associated with the BFNE, whereas the spatial imagery scale of the VISQ was significantly negatively associated with the BFNE following Step 2 of the analysis. Moreover, the interactions of the VISQ object scale × EC scale and the VISQ spatial scale × EC scale were significant. The results of a simple slope analysis indicated that the VISQ object scale was positively associated with the BFNE when scores on the EC scale were medium (*B* = 0.18, β = 0.18, *t* = 2.00, *p* < 0.05) and low (*B* = 0.46, β = 0.45, *t* = 3.60, *p* < 0.001), whereas the VISQ object scale was not significantly associated with the BFNE when scores on the EC scale were high (*B* = -0.10, β = -0.10, *t* = -0.73, *p* = 0.47; **Figure 1A**). The analysis also indicated that the VISQ spatial scale was negatively associated with the BFNE when scores on the EC scale were medium (*B* = -0.26, β = -0.27, *t* = -3.93, *p* < 0.001), and low (*B* = -0.40, β = -0.42, *t* = -4.03, *p* < 0.001), whereas the VISQ spatial scale was not significantly associated with the BFNE when scores on the EC scale were high (*B* = -0.11, β = -0.12, *t* = -1.25, *p* = 0.21; **Figure 1B**). Multicollinearity was checked by calculating the variance inflation factor (VIF), and all VIF values were less than 3. When gender and age were entered in Step 1 as the covariates, the mental imagery scales and EC in Step 2, and the interactions between mental imagery and EC in Step 3, the results were the same as above.

**Table 2:** The hierarchical regression analysis for mental imagery scales and EC predicting BFNE.

	Step 1						Step 2					
	*B*	*SE B*	β	*t*	Δ*R*^2^	*Adj. R*^2^	*B*	*SE B*	β	*t*	Δ*R*^2^	*Adj. R*^2^
VVIQ	-0.027	0.075	-0.026	-0.363	0.103^∗∗^	0.078	-0.005	0.074	-0.005	-0.066	0.069^∗∗^	0.130
VVQ visualizer	0.129	0.144	0.075	0.897			0.105	0.142	0.061	0.740		
VVQ verbalizer	0.044	0.098	0.031	0.447			0.052	0.099	0.037	0.524		
VISQ object	0.207	0.093	0.200	2.235^∗^			0.181	0.090	0.175	2.000^∗^		
VISQ spatial	-0.234	0.066	-0.247	-3.526^∗∗^			-0.257	0.065	-0.271	-3.934^∗∗^		
EC	-0.088	0.047	-0.123	-1.878			-0.057	0.048	-0.080	-1.198		
VVIQ × EC							0.011	0.006	0.138	1.783		
VVQ visualizer × EC							0.009	0.012	0.061	0.736		
VVQ verbalizer × EC							-0.013	0.010	-0.101	-1.377		
VISQ object × EC							-0.024	0.008	-0.286	-2.932^∗∗^		
VISQ spatial × EC							0.012	0.006	0.147	2.076^∗^		

^∗^*p < 0.05, ^∗∗^*p* < 0.01*.

## Discussion

The present study investigated the relationship between social anxiety and mental imagery ability, and the interactive effects of EC with mental imagery on social anxiety. A correlation analysis showed that social anxiety was positively associated with a preference for visual mental images and object mental imagery, and it was negatively associated with spatial mental imagery. A hierarchical regression analysis of mental imagery showed that only object mental imagery and spatial mental imagery predicted the degree of social anxiety positively and negatively, respectively. Moreover, scores on the EC scale moderated the effects of these mental imagery scales on social anxiety. High EC could suppress the maladaptive effects of high object mental imagery and low spatial mental imagery on social anxiety.

Consistent with the hypothesis, social anxiety was associated with visual imagery, but not with verbal processing. Because verbal processing could attenuate emotional arousal (Holmes and Mathews, 2005, 2010), verbal representations might be useful to calm down socially anxious individuals in social situations. However, such individuals generally use visual processing and tend to focus on emotional processing. Verbal representations also attenuate the believability of images (Holmes and Mathews, 2010). Therefore, visual processing in social anxiety might lead to a high likelihood of negatively imagined experiences.

Additionally, the present findings suggested that not all features of visual imagery were correlated with social anxiety. The vividness of mental images was not associated with social anxiety. The present result was not consistent with those of previous studies, which have shown that anxious individuals had more vivid imagery for neutral or negative situations (Stöber, 2000; Moore, 2002; Morina et al., 2011). There are two differences between the present and previous findings. First, the imagined situations were different. In the present study, mental imagery for “neutral” situations was measured, whereas vivid imagery in previous studies was shown for “negative” events (e.g., rejection from someone) (Stöber, 2000; Morina et al., 2011). Second, the types of “anxiety” were different. Although Moore (2002) showed high imagery vividness for neutral scenes in “anxiety,” participants had generalized anxiety disorder—worrying about non-specific events—and not social anxiety disorder. “Social anxiety,” on the other hand, is related to the fear of “social” situations and interaction with other people. Considering these differences, it is possible that, in “natural” scenes, socially anxious individuals do not necessarily generate vivid mental imagery, whereas in “social” situations, they might exhibit vivid imagery. The different functions of neutral and social information in social anxiety were observed in attentional functions (Moriya and Tanno, 2010; Yoon et al., 2015). Socially anxious individuals exhibited broadened attentional scope for neutral stimuli (e.g., letters) to process peripheral stimuli, whereas they showed narrowed attention for social stimuli (e.g., faces). It is possible that mental imagery works in a manner similar to attentional functions. Because mental imagery also has a limited capacity (Keogh and Pearson, 2017), socially anxious individuals broaden their internal attention to mental images for neutral information, which might reduce the vividness of images; whereas, narrowed internal attention for social information could help visualize the information much more vividly. Further studies need to manipulate situations to measure imagery vividness in social anxiety.

The finding indicating a preference for processing visual mental images, especially object mental imagery in social anxiety, was consistent with the hypothesis. Previous studies have shown that high object mental imagery is related to a preference for visualizing scenes when people listen to dialogs (Blajenkova et al., 2006; Kawahara and Matsuoka, 2009). Moreover, when people listen to negative dialogs, visually negative mental images of themselves elicit more anxiety in comparison with the images generated via verbal processing (Holmes and Mathews, 2005). This indicates that socially anxious individuals with object imagery might prefer to imagine situations visually, which increases their anxiety.

Although spatial imagery is a subsystem of visual imagery, the present results showed a negative correlation between social anxiety and spatial imagery. Based on cognitive theories (Clark and Wells, 1995; Rapee and Heimberg, 1997), socially anxious individuals take the observer perspective. Therefore, it was hypothesized that social anxiety was positively correlated with spatial imagery, which is associated with perspective transformation. The present results suggested that socially anxious individuals involuntarily take the observer perspective, whereas they have difficulty in voluntarily changing their own perspective. Kaltner and Jansen (2014) conducted the mental rotation test, which is associated with voluntary spatial imagery, and showed poor mental-rotation performance in highly anxious individuals. They insisted that impoverished prefrontal attentional control in anxiety delayed the performance in highly anxious individuals because mental rotation involves the active manipulation of visual representation, which is more a controlled process of voluntary attention. Possibly, because social anxiety is also related to poor attentional control (Moriya and Tanno, 2008), spatial imagery was negatively correlated with social anxiety in the present study.

Effortful control is an important factor for protecting against increased social anxiety. Consistent with the findings of previous studies (Moriya and Tanno, 2008), the present study revealed that scores on the EC scale were negatively correlated with social anxiety. Moreover, EC was also effective in preventing an increase in social anxiety when individuals exhibited maladaptive processing of mental images. Although high object imagery and low spatial imagery were associated with high social anxiety, these associations were not significant for individuals with high EC. While previous studies have shown that anxiety symptoms were inhibited in anxious individuals with high EC (Stevens et al., 2015), the present results showed that EC also mitigated the impact of maladaptive mental processing in social anxiety. The present results also suggest that high object imagery and low spatial imagery might be maladaptive for social anxiety when individuals cannot control their attention. If so, why is EC effective for protecting against an increase in social anxiety? One possibility is that socially anxious individuals with high EC exhibit decreased processing of visual mental images and tend to use verbal strategies for mental images. The present result showed a positive correlation between EC and the verbalizer scale of the VVQ. Because verbal processing of mental images decreases emotional effects (Holmes and Mathews, 2005), individuals with high EC could inhibit the increase in social anxiety.

There are some limitations in the present study. First, the accuracy of participants’ responses is unclear due to the use of self-report scales alone. While socially anxious individuals think that they prefer visual mental images, it is still unclear what they imagine in real situations. Some previous studies used a behavioral measure of imagery in experiments and showed an impaired ability to generate visual mental images in social anxiety (Morrison et al., 2011; Amir et al., 2012). Therefore, future studies should use objective, non-self-report experiments to investigate these mental processes accurately. Second, although some results of the correlations and regression were statistically significant, the effects were small. It is possible that the effects of mental imagery on social anxiety are subtle. If extremely highly socially anxious individuals are selected and are compared with controls, the effect size might increase. Another possibility is that apprehension about others’ evaluations, which was measured in the present study as part of social anxiety, was subtly influenced by mental imagery, whereas other features of social anxiety, such as physical symptoms and avoidant behavior, were influenced more. By using several scales to assess social anxiety and mental images, it would be possible to ascertain the aspects of social anxiety that are associated with mental imagery abilities. Third, it is possible that the present predictive model was overfitting because of several variables. To avoid overfitting, future studies should reduce confounders and replicate the present results. Fourth, because the participants comprised a non-clinical sample of students, it is not clear whether the present results are applicable to a clinical population. Considering the social anxiety continuum (Kollman et al., 2006; Ruscio, 2010), the present results might also be observed in social anxiety disorder. Finally, because few studies have investigated the mental imagery for neutral scenes in social anxiety, more studies are necessary to reveal the robustness of and the mechanisms underlying the present results.

In sum, the present study showed that social anxiety was positively related to a preference for visual mental images, especially object imagery. Socially anxious individuals might prefer to construct pictorial images of individual objects in natural scenes, which might increase their anxiety. However, even for individuals who exhibit these features of mental imagery, EC could inhibit the increase in social anxiety. Individuals with low EC and high object imagery or low spatial imagery showed high social anxiety. These results regarding the relationships between mental imagery and social anxiety might be useful to develop efficient mental techniques for treating social anxiety disorder.

## Ethics Statement

This study was carried out in accordance with the recommendations of Graduate School of Psychology, Ethics Committee.

## Author Contributions

The author confirms being the sole contributor of this work and approved it for publication.

## Conflict of Interest Statement

The author declares that the research was conducted in the absence of any commercial or financial relationships that could be construed as a potential conflict of interest.
